# Major transitions in sociocultural evolution

**DOI:** 10.1017/ehs.2025.10021

**Published:** 2025-10-10

**Authors:** Arsham Nejad Kourki

**Affiliations:** 1 Theoretical Biology Lab, The Francis Crick Institute, London, UK; 2 Department of History and Philosophy of Science, University of Cambridge, Cambridge, UK

**Keywords:** Evolutionary Transitions in Individuality, Major Evolutionary Transitions, Cultural Group Selection, Sociocultural Evolution

## Abstract

Recent years have seen growing interest in applying the Evolutionary Transitions in Individuality (ETI) framework to human sociocultural evolution. Proponents argue that human societies exhibit features – such as multilevel organization, cooperation, and division of labour – sufficiently analogous to biological ETIs to warrant theoretical extension. This paper critically assesses such claims and argues that they rest on a fundamental misapplication of the ETI framework. Drawing on recent work in cultural evolution, I show that sociocultural systems typically lack the core conditions required for an ETI, including autonomous reproduction at the group level and the operation of natural selection in the reproductive mode. Attempts to relax these criteria risk undermining the coherence of the framework itself. I conclude that although the broader framework of Major Evolutionary Transitions may still have value for understanding sociocultural change, the specific explanatory structure of ETI theory does not transfer.

## Graphical abstract

**Figure:**
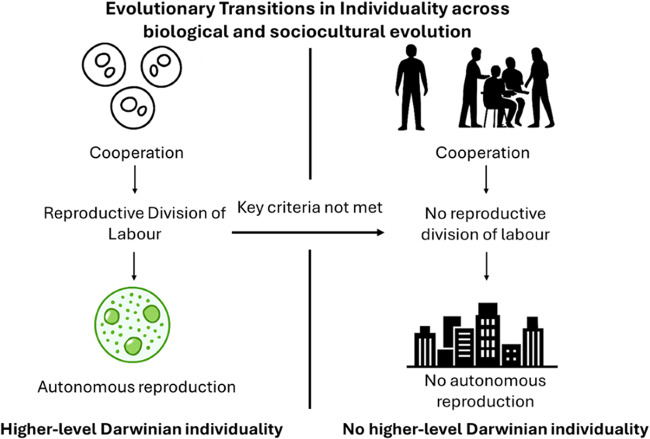

## Social media summary

Why human sociocultural evolution may involve major transitions, but not evolutionary transitions in individuality.

## 1. Introduction

Human societies exhibit striking forms of complexity, coordination, and multilevel organization. These features have long invited comparison to biological organisms – seen most famously in metaphors like the ‘social organism’ and the ‘body politic’ – but in recent decades, they have also motivated efforts to extend evolutionary theory itself into the sociocultural domain. One especially ambitious strand of this work draws on the biological literature on Major Evolutionary Transitions (METs), and in particular on the Evolutionary Transitions in Individuality (ETI) framework, to propose that human sociocultural evolution may have involved comparable transitions. According to these proposals, human groups may in some sense function as higher-level evolutionary individuals, shaped by selective pressures acting at the collective level.

Such ideas have gained renewed attention in recent years, especially in the wake of formal models of cultural group selection (CGS), and the publication of dedicated volumes and journal issues aiming to reframe human history as a series of cultural or societal ETIs. These accounts often begin from a natural analogy: just as multicellular organisms evolved from unicellular ancestors, human societies may have evolved from loosely connected individuals into cohesive and functionally differentiated collectives. The suggestion is that by applying the theoretical tools of the ETI framework – originally developed to explain the emergence of new units of selection in biological evolution – we can gain insight into the large-scale cooperative structures and historical transitions that characterize human social life.

This paper argues that although the analogy is intuitive and heuristically rich, the direct application of the ETI framework to sociocultural evolution is deeply problematic. I show that the core explanatory conditions of the ETI approach – especially those pertaining to reproduction and individuality – are not met in the sociocultural domain, and that attempts to reinterpret or weaken these conditions risk undermining the framework’s conceptual coherence. I suggest that a more philosophically cautious and domain-sensitive strategy is needed, one that acknowledges both the potential relevance and the limits of biological theorizing in this context.

## 2. Evolutionary Transitions in individuality in sociocultural evolution

### 2.1. The ETI approach

The ETI approach is a theoretical framework developed in evolutionary biology to explain a distinctive class of macroevolutionary events: those in which new kinds of biological individuals emerge from collectives of pre-existing ones. The canonical cases include the evolution of chromosomes from independent genes, eukaryotic cells from prokaryotes, multicellular organisms from unicellular ancestors, and eusocial colonies from solitary insects (Clarke, 2014; Herron, 2021; Maynard Smith & Szathmáry, 1995; Michod, 2000; Michod & Herron, 2006; Michod & Nedelcu, 2003; Okasha, 2006, 2022; Shelton & Michod, 2020). In each of these cases, formerly autonomous units came to form cohesive collectives that acted as integrated evolutionary individuals – entities upon which natural selection could operate as a whole. What makes these transitions ‘in individuality’ is not simply the emergence of cooperation or complexity, but the transformation of evolutionary structure: selection begins to act primarily at a higher level of organization, whereas lower-level entities lose the capacity for independent reproduction. This typically involves the evolution of mechanisms that align interests among parts (e.g. bottlenecks, policing mechanisms, or reproductive division of labour), enabling the collective to function as a coherent, reproducing whole (Godfrey-Smith, 2009; Herron, 2021). ETI theory thus provides a focused answer to a foundational question: how do new levels of biological organisation – genuine Darwinian individuals – arise in evolutionary history? In doing so, it offers a precise and coherent criterion for identifying certain major evolutionary transitions: namely, those that involve the emergence of new units of selection through the reorganization of fitness.

The ETI approach can be seen as a particular refinement within the broader framework of METs. The MET framework, introduced by Maynard Smith and Szathmáry (1995), aimed to identify common features across major episodes of innovation in evolutionary history, such as changes in the way information is stored and transmitted, or the emergence of new organizational levels. Wheres MET theory is broader and somewhat more heterogeneous in scope, the ETI approach carves out a theoretically precise subset of these events – those specifically involving the emergence of new evolutionary individuals. In doing so, it preserves the explanatory ambition of the MET framework while offering a more narrowly defined and testable set of conditions.

In recent years, researchers have begun to explore whether the ETI framework might also be fruitfully applied to sociocultural evolution. After all, human societies exhibit many of the surface features associated with biological ETIs: multilevel organization, coordination among parts, division of labour, and sometimes even strong conformity or norm enforcement. These parallels have inspired a range of proposals suggesting that human societies may themselves constitute – or at least approximate – higher-level evolutionary individuals. The appeal is understandable: ETI theory offers a ready-made toolkit for thinking about the emergence of large-scale, cohesive social structures.

Yet as we shall see, the apparent similarities between biological and sociocultural organization can obscure deeper differences. The explanatory power of ETI theory depends on the satisfaction of a stringent set of biological conditions – notably, autonomous reproduction and selection operating on a Darwinian population of higher-level entities. Whether such conditions are meaningfully instantiated in the sociocultural domain is far from obvious. The remainder of this paper critically assesses recent attempts to apply the ETI framework to human evolution and argues that a more careful, domain-sensitive approach is needed.

### 2.2. Applying the ETI approach

There is a natural analogy to be drawn between human societies and biological organisms or superorganisms, and it has been drawn many times in our intellectual history. Some of the most recognizable ones include Plato’s *Republic*, Hobbes’ *Leviathan* (Hobbes, 1651), Spencer’s *Social Organism* (Spencer, 1860), Durkheim’s *Division of Labour* (Durkheim, 1893), and Parsons’ *The Structure of Social Action* (Parsons, 1937). I can entertain little doubt that a proper survey of global intellectual history will reveal countless other examples. The common theme running through these analogies tends to be the recognition that in both human societies and biological (super)organisms the constituent units make up a collective that itself behaves, in some ways, remarkably similarly to the constituent units. It thus seems quite natural to then consider the question of *how* the higher-level entities (the collectives) emerge from the lower-level, constituent units. This, in other words, is a question about the *transition to a higher level of organization* and is the fundamental driving force behind efforts to apply the ETI approach specifically, and the MET framework more generally, to sociocultural evolution. It marks an intuition: multilevel organization, especially when accompanied by phenomena such as division of labour, ought to be explained via the explanatory apparatus of the ETI approach (or a closely related approach falling within the broader MET framework), whether in the biological domain or the sociocultural. My central aim here is to show that this intuition is misguided but redeemable. Let us first look at some of its explicit articulations in recent years.

One interesting example of attempting to bring the MET framework to sociocultural evolution is Geoffrey Hodgson and Thorbjørn Knudsen’s *Darwin’s Conjecture: The Search for General Principles of Social and Economic Evolution* (Hodgson & Knudsen, 2010). The authors argue that the principles of variation, inheritance, and selection can illuminate how complex social structures and institutions emerge, adapt, and persist over time. Crucially, they point out that (1) sociocultural systems (e.g. tribes, institutions), like biological ones, are organized across multiple levels of organization and that (2) the emergence of these levels can be explained in terms of the emergence of new systems of replication (e.g. the emergence of habits, customs, and laws), framing this in terms of the distinction due to Hull (1980) between interactors and replicators – in this case things like institutions and laws, respectively (although the connection between the replicator/interactor dichotomy and the METs goes back to Maynard Smith and Szathmáry’s original account).

In a somewhat similar vein, Peter Turchin suggests in *Ultrasociety: How 10,000 Years of War Made Humans the Greatest Cooperators on Earth* (Turchin, 2016) that human societies have undergone a number of METs since the emergence of civilizations in the Late Neolithic. Although Turchin draws mainly on ecological modelling and life-history theory in this work as well as his earlier work (Turchin & Nefedov, 2009), there is a strong and often explicit element of talk about METs running in the book as well (he has also personally communicated to me his thinking on this matter in these terms). Similarly to other such expositions, Turchin takes the increasingly large scale of human societies as enabled by increasingly greater degrees of human cooperation. What makes his account distinct, though, is his reliance on mathematical models and a large historical data set to argue that these transitions were primarily driven by the rise of novel military technologies such as chariots and firearms.

A number of more recent papers focus more directly on applying the ETI approach in this domain. For example, Timothy Waring and Zachary Wood (2021) discuss an ‘Evolutionary Transition in Inheritance and Individuality’ as involving a transition from genetic to cultural inheritance (which is akin to Maynard Smith and Szathmáry’s transition from genetic to linguistic mode of information transfer) and a resulting transition in individuality from cultural individuals to cultural superorganisms (i.e. societies), thereby also retaining both informational and structural aspects of METs. In addition, the *Philosophical Transactions of the Royal Society B* published a special issue titled *Human socio-cultural evolution in light of evolutionary transitions* in March 2023 (Carmel et al., 2023). While the theme of taking ETIs to the sociocultural domain runs throughout the special issue, I will only highlight a few papers here that are more relevant for the purpose of this paper.

First, Davison and Michod (2023) argue that the key steps in an ETI – group formation (with increases in group size), cooperation, conflict and conflict mediation, division of labour and the export of fitness to the group level and the inheritance and heritability of group-level traits – based on a close analysis of the evolution of multicellularity in the volvocines algae, are also to be found in human evolution from our last common ancestor with *Pan* through to the transition from the Oldowan to the Acheulean. It is particularly interesting and relevant here that they identify a transfer of fitness in cultural evolution despite the virtual absence of reproductive division of labour. As one reviewer has pointed out, one might contend that reproductive division of labour is not, strictly speaking, absent in human societies, because women tend to devote a larger proportion of their lives to rearing offspring and men tend to funnel more resources into it. This is nevertheless hardly comparable to what we see in the case of germlines or hive queens. I will return to this point later when criticising the application of the ETI to this domain.

Second, Yohay Carmel (2023) poses the following question: *Human societal development: is it an evolutionary transition in individuality?* He uses three criteria – size, specialization, and inseparability – based on an earlier work (Carmel & Shavit, 2020) to evaluate potential ETIs in recent human sociocultural evolution. Size refers to the number of lower-level units; specialization refers to differentiated roles among them (both reproductive and non-reproductive); and inseparability refers to the inability of those units to persist independently of the larger whole. Size and non-reproductive specialization have undeniably increased across human history, whereas reproductive specialization remains absent. Inseparability, however, is more complex. Humans today are in many ways more dependent on one another than ever before: subsistence, technology, and institutions are increasingly distributed across social roles that no individual or small group could replicate alone. In this sense, inseparability may indeed capture an important feature of sociocultural evolution and may even help explain the emergence of higher-level social entities. Yet crucially, inseparability of lower-level units does not entail reproductive autonomy at the higher level. Societies may become internally interdependent without thereby becoming autonomous reproducers in the Darwinian sense. Based on these criteria, he identifies six main transitions: (1) from forager bands to early agricultural settlements in the Levant, (2) the emergence of the first small cities such as Jericho and Çatalhöyük in the neolithic in the Levant and Anatolia, (3) the emergence of the first city-states in Mesopotamia and the appearance of writing, (4) the emergence of the first kingdoms starting with unified Egypt and later the Akkadian Empire, (5) the emergence of true empires starting with Achaemenid Persia followed by others such as the Roman Empire, China, and the Islamic Caliphates, and, finally and more tentatively, (6) the emergence of ‘the global network as a single coherent and interconnected social and economic unit’.

Third, Andersson and Czárán (2023) propose the social protocell hypothesis, which conceptualizes early human groups as prototypical ‘social cells’ exhibiting characteristics analogous to biological protocells. These early human collectives are posited to have formed cohesive, bounded units that could interact with their environment and engage in competition with other groups, thus undergoing selection at the level of the group. Andersson and Czárán argue that such social protocells represent an essential stage in the evolutionary trajectory toward greater societal complexity. Although lacking physical boundaries akin to cellular membranes, these groups maintained cohesion through cultural norms and social structures that functioned as analogous containment mechanisms, ensuring group integrity and facilitating interactions both within and between groups. This hypothesized stage is seen as a necessary precursor to more pronounced transitions involving division of labour, increasing specialization, and the inheritance of group-level traits. In a later paper, Andersson and Tennie trace the evolution of social protocells back to our last common ancestor with *Pan* (Andersson & Tennie, 2023).

Finally, Daniel McShea (2023) takes an interesting turn by criticising the applicability of the ETI approach to sociocultural evolution, citing four reasons:

(i) The foundation of the major transitions is hierarchy, but the cross-cutting interactions in human societies undermine hierarchical structure. (ii) Natural selection operates in three modes – stability, growth and reproductive success – and only the third produces the complex adaptations seen in fully individuated higher levels. But human societies probably evolve mainly in the stability and growth modes. (iii) Highly individuated entities are marked by division of labour and commitment to morphological differentiation, but in humans differentiation is mostly behavioural and mostly reversible. (iv) As higher-level individuals arise, selection drains complexity, drains parts, from lower-level individuals. But there is little evidence of a drain in humans.

My criticism of applying the ETI approach in sociocultural evolution is essentially the same as the second reason. But one might notice that this reason seems *prima facie* strongly at odds with the whole literature on cultural group selection (CGS), which relies heavily on natural selection being responsible for a whole range of, and perhaps all, cultural adaptations. To address this, I will now very briefly discuss the literature on CGS and highlight that it is the conceptual foundation upon which attempts at applying the ETI approach to sociocultural evolution are built. In the following section, I will go into McShea’s criticism (ii) in more detail and supplement it with a more recent argument by Sterelny (2024), and will furthermore argue that this criticism is in fact the most critical of them all if we consider the clearest and most precise formulation of the ETI approach to date (Herron, 2021). Finally for Section 3, we shall revisit the recent applications of the ETI approach to sociocultural evolution.

### 2.3. Cultural group selection and ETI

CGS represents one attempt to understand how cultural traits in human societies evolve analogously to biological traits, primarily through intergroup competition. Within this framework, cultural traits – including practices, norms, and technologies – are thought to undergo selection at the group level, particularly when they contribute to the cohesion and cooperation that enhance collective fitness. Here, advantageous cultural traits promote a form of social organization that makes groups more competitive, enabling them to outlast or displace less cooperative groups. Thus, CGS posits that cohesive and cooperative groups tend to outcompete less organised ones, suggesting a mechanism by which cultural practices favouring group welfare over individual gain persist.

Recent work articulates the processes by which CGS might operate, focusing on social learning and norm enforcement as essential for the stability of cooperative traits. For example, Richerson and Boyd (2008) emphasize the role of conformist transmission – where individuals adopt the most common practices within a group – and the punishment of norm violators. According to this theory, these mechanisms serve to stabilize group-beneficial norms, reinforcing cooperation and cohesion. Henrich (2016) furthers this view, proposing that cultural evolution has moulded human psychology to support large-scale cooperation. According to Henrich, CGS has not only facilitated group cohesion but also driven the development of complex societies, as groups with well-integrated norms and collaborative strategies have historically prevailed over those lacking such traits.

Not all outcomes of CGS, however, invite a major transitions interpretation. Explaining the dynamics of small-scale groups – such as forager bands – may demonstrate cultural competition but does not in itself warrant treating these groups as new units of organization in the ETI sense. What makes the analogy with ETIs more compelling is when CGS is invoked to explain the emergence of large, stratified, and multilevel societies, where cohesion and norm enforcement sustain collective structures far beyond face-to-face groups.

Now, recall that the ETI approach attempts to explain the emergence of higher levels of organization in terms of the evolution by natural selection of traits which promote cooperation and cohesion at that level. The tight conceptual link between CGS and ETI in the sociocultural domain should therefore be quite obvious once we observe that some sort of group selection is required for there to be an ETI. In this sense, CGS provides the conceptual foundation for applying the ETI approach to this domain. This is because it makes it seem plausible that indeed we do find natural selection acting at multiple levels of sociocultural organization, and that the higher levels are therefore themselves the result of this multilevel selection process. In fact, much of the work in the latter area implicitly or explicitly invokes CGS (Davison & Michod, 2023; Davison et al., 2021; Shelton & Michod, 2020; Townsend et al., 2023; Waring & Wood, 2021). Crucially, the same set of criticisms, spearheaded by McShea’s reason (ii) to be sceptical pointed out above, apply to both.

## 3. Inapplicability of the ETI approach

### 3.1. Different kinds of selection

There is a reciprocal relationship between the operability of natural selection at a given level of organization and the emergence of reproductive autonomy at that level of organization. In other words, *because natural selection paradigmatically acts on Darwinian populations, units occupying a level of organization need to be autonomous reproducers/members of a Darwinian population for selection to act at that level; i.e. for them to be units of selection*. Here, I take units of selection to be equivalent to evolutionary individuals (see Clarke, 2014; Herron, 2021). This is worth the clarification because one might also construe units of selection more broadly to include ‘trait groups’ (see Wilson & Sober, 1998). Furthermore, reproductive autonomy at a level of organization typically comes with loss of reproductive autonomy at the lower level of organization, which is itself a corollary of the evolution of reproductive division of labour (e.g. germline vs soma, queen vs workers), although some models of cultural group selection – such as Boyd and Richerson’s equilibrium selection models – illustrate that higher-level dynamics need not always entail such suppression. So, the key question is this: do higher-level entities in the sociocultural domain comprise Darwinian populations? In other words, are these entities typically units of selection? *Are they autonomous reproducers?* And a secondary question is: do we find reproductive division of labour in human societies? (See Table 1 for an overall comparison.)

**Table 1.** Key differences between biological and sociocultural systems relevant to the applicability of the ETI framework

Feature	Biological domain	Sociocultural domain
Units of Selection	Autonomous reproducers (e.g. cells, organisms)	Rare or absent; social groups typically not reproducers
Dominant mode of selection	Reproductive (natural selection in Darwinian populations)	Growth and stability modes; little reproductive selection
Reproductive division of labour	Present (e.g. germ–soma, queen–worker)	Weak, if at all present (stronger maternal care)
Inheritance mechanism	Genetic, vertical, high fidelity	Cultural, often horizontal, hybrid, low fidelity
Cohesion mechanisms	Structural and developmental (e.g. membranes, ontogeny)	Norms, institutions, shared belief systems
Organizational hierarchy	Nested, part–whole (cells → tissues → organs → organisms)	Overlapping, cross-cutting (e.g. kinship, institutions, polities)
Rate of reproduction/variation	Fast enough for iterative selection	Very slow or absent (e.g. few ‘daughter societies’)
Eligibility for ETI framework	High	Low (fails core conditions)

I will return to the second question later. For now, let us revisit McShea’s reason (ii) to be sceptical of ETIs in sociocultural evolution in a bit more detail. First off, McShea brings up a distinction between three kinds of selection originally formulated by L. Van Valen (1976): survival, growth, and reproductive modes (see also Bouchard, 2008; L. M. Van Valen, 1989; McShea & Simpson, 2011; Simpson, 2012, 2013). The reproductive mode is what Godfrey-Smith characterizes as what happens in a paradigmatic Darwinian population, and is what people like Lewontin or Maynard Smith typically meant when talking about the conditions for natural selection to operate in a population (Lewontin, 1970; Maynard Smith, 1988). So, it requires the existence of autonomous reproducers. In McShea’s words:

In reproductive mode, individuals with a greater propensity to survive and reproduce leave more surviving offspring, and their descendants replace those with a lower propensity. This is the conventional understanding of selection.

But growth and survival modes are different, and successively less demanding:

Selection in growth mode is different. A clonal aspen variant spreading on a hillside … is fitter than – and will replace – other variants if it simply grows faster than they do. Likewise a coral variant is fitter than its competitors if it is able to channel more of its surplus metabolic energy … into growth. … In pure growth mode, fitness is fatness. For selection in this mode … fitness is success in the competition for resources, more specifically for energy (L. Van Valen, 1976). …

Finally consider persistence mode (Bouchard, 2008). A protist might be fitter than its competitors if it is able to encyst for long periods of time and thereby survive longer. Its competitors go extinct, perhaps by chance, as all species inevitably do, while the fitter individual persists.

McShea then goes on to make not only the uncontroversial claim that the reproductive mode (and not growth mode) is the one responsible for the evolution of complex adaptations, but also the claim that this mode of selection is virtually absent in the sociocultural domain, barring some possible exceptions:

So in what selective mode do human societies evolve? It is hard to see human societies as reproducers. They do reproduce sometimes … but for the reproductive mode to generate complex adaptations, ideally reproduction would generate larger numbers of offspring. Each human society would have to produce many daughter societies. And it seems hard to argue that that happens very often. … Rather societies seem to operate predominantly in growth mode, by spreading. … [I]t is clear that the rate of reproduction or growth is very slow, and that human societies are not able to take advantage of the massively parallel accumulation of variation that is so essential to the production of complex adaptation. So … it seems unlikely that human societies can be highly individuated. The process needed to drive this transformation would simply have been too slow, the intensity of selection favouring it too weak.

Cases from biology show that growth can contribute to fitness when coupled with reproduction. For instance, clonal growth in plants can enhance genet fitness both through persistence and increased seed production (Pan & Price, 2001). By contrast, in human societies growth is largely decoupled from reproduction, which is precisely why its explanatory power for adaptive evolution is more limited. And though this is targeted as a criticism of applying ETI to sociocultural evolution, it is simultaneously a criticism of CGS. But, as McShea admits:

Again, these judgements are impressionistic. For objective assessments, we would need not only data on rates of reproduction, growth and survivorship of human societies, but the equivalent data for organisms that are thought to have undergone major transitions.

Which brings me to Sterelny’s recent criticism of the role of selection in sociocultural evolution more generally, which includes a criticism of CGS more specifically (Sterelny, 2024); for a broader discussion, see Lewens (2024). Although Sterelny acknowledges the importance of multigenerational accumulation of adaptive information – a cornerstone of cultural evolutionary theory – he expresses scepticism about the role of natural selection in this. He argues that key conditions required for selection on individual cultural traits are absent in human societies. Drawing on ethnographic studies, Sterelny highlights that human learning typically involves a mix of social and environmental inputs, often through what he calls ‘hybrid learning’, rather than high-fidelity copying from a single model. This reliance on multiple learning sources dilutes the impact of any single individual’s cultural traits on the population, which limits the efficacy of selection at the individual level.

Sterelny also questions the assumption that cultural transmission is lineage-based. In traditional Darwinian evolution, adaptations spread through parent–offspring inheritance, establishing clear lineage paths. In human cultural contexts, however, individuals learn from a variety of community members, not just direct ancestors. This diffusion means that specific cultural variants lack a clear ‘parent’, and without distinct lineages, traits cannot consistently be passed down across generations in a manner that selection would act upon.

He concludes that individual-based selection might apply in some narrow cases where transmission fidelity and lineage structure are unusually high (Migliano et al., 2017; Migliano & Vinicius, 2022), but these conditions are rare. Thus, he casts doubt on individual-level selection as a primary driver of complex human adaptations, suggesting that cultural evolution in humans operates largely outside of strict Darwinian mechanisms.

But what about CGS? Sterelny does not hold back:

This process may well have played some role in explaining human adaptation. But this role was probably minor. First, selection acts most effectively on large populations. … [F]orager communities interact with, and hence are potentially in competition with, few other communities. … Moreover, on the most intuitive ways of understanding group fitness (determined by community lifespan and fecundity), generational turn-over was slow, and the clock of evolution ticked slowly. … Yet populations seemed to adapt relatively rapidly to new conditions. … There are ways of understanding group fitness that avoids this problem; for example, counting local population increase and in-migration as aspects of group fitness. But these are more naturally seen as growth rather than reproduction: large trees are not thereby fitter for having more cells.

We can see here that an important part of the reasoning is virtually identical to McShea’s: that the only kind of natural selection at higher levels of organization that we can find in sociocultural evolution is growth selection, and growth selection has nothing like the explanatory power of reproductive selection. Sterelny follows this up by what he takes to be a more serious set of problems, namely ‘the identification of competing groups’. The gist of the matter is that there are two candidate kinds of competing groups in forager societies, namely residential ones and ethnolinguistic ones, and neither of these two are actually suitable candidates for natural selection; the former because there is little selectable variation among them, and the latter because they do not genuinely act as units. Sterelny closes his paper with the suggestion that in the absence of natural selection as the explanans of adaptive sociocultural evolution we can instead turn to other processes such as niche construction, hybrid learning, and ecological inheritance.

Two points of clarification before we move on. Firstly, by ‘residential groups’, Sterelny refers to the small, fluid forager bands – typically a few dozen individuals living together at a given time – that form the most immediate units of daily life. These are cohesive in the short term but highly permeable, with frequent movement of individuals and families between bands. Secondly, it is true that sedentary village societies present somewhat larger and more bounded groups, and in this respect Sterelny’s point is weaker there (Seabright, 2004). Yet the contrast should not be overstated. Archaeological evidence shows that even the earliest permanent settlements were permeable to the movement of artefacts and ideas across wide distances, whereas forager bands themselves were not without intergroup competition.

At this point, a proponent of applying ETI to sociocultural evolution might object that my understanding of this approach is too narrow. Perhaps the emergence of a higher-level individual does not have to be understood as the emergence of autonomous reproducers – perhaps all that is needed is the emergence of traits that in turn explain the emergence of somewhat cohesive entities at higher levels of sociocultural organization. After all, it does seem like that is what many present authors are trying to claim. However, this is a problematic objection. Firstly, as we have seen, the explanatory power of the ETI approach is rooted precisely in its reliance on autonomous reproducers. Once again, autonomous reproduction is what grants fitness to (and makes something a) unit of selection, which in turn also explains how it goes on to accumulate adaptations. Secondly and relatedly, such an objection amounts to subscribing to a vague formulation of the ETI approach that lacks the aforementioned grounding and explanatory power, as opposed to the precise and powerful formulation made recently by Herron (2021). But perhaps a vague formulation is all we need, and that we should allow the prospective productivity of applying this approach to sociocultural evolution speak for itself. The main aim of the final subsection before the conclusion is to respond to this argument. But for now, let us revisit two of the recent papers on applying the ETI approach to sociocultural evolution mentioned above to see how they fare with respect to the present criticism.

### 3.2. Cultural traditions and socionts

In this subsection, I will only go through two of the works mentioned above, because unlike many others they both attempt to go around the inapplicability problem in interesting ways.

First, the paper by Richard Michod – perhaps the main architect of the ETI approach – and Dinah Davison (Davison & Michod, 2023). As mentioned above, they identify key stages of ETIs in sociocultural evolution and conclude that this gives sufficient reason to believe that there has indeed been a transfer of fitness and thus an ETI in sociocultural evolution, despite recognizing the absence of reproductive division of labour there. This gives rise to a conundrum: if there are no reproductive division of labour and, concurrently, no clear autonomous reproduction at the higher level, then what are the bearers of fitness at the higher level? Davison and Michod resolve this problem by ascribing fitness and division of labour not to social groups, but to *integrated groups of cultural traditions*:

Although reproductive division of labour (specialization on transmission and/or persistence) on may not exist in culture, division of labour more generally is implicit in our discussion of tradition groups in hominin culture … While reproductive division of labour may not exist among the traditions that make up a large game hunting system, there still are likely trade-offs between the functions of traditions. Hunting large game in *Homo* is an example of such a goal and provides examples of division of labour in a group of traditions. As described previously, traditions regarding obtaining and processing tools and meat must all be present and expressed through the behaviour of hominins for a hunt to be completed successfully. Each of these traditions is part of the larger system of traditions and many component traditions only make sense in the context of the other components of that system (Cavalli-Sforza & Feldman, 1981; Nettle, 2020).

This move effectively dodges the full weight of the criticism discussed above because it avoids claiming that human social groups can undergo paradigmatic natural selection, and it retains some of the explanatory value of the ETI approach in sociocultural evolution. But it is nonetheless a strange move, not least because it is hard to square integrated groups of cultural traditions as some kind of individual. Cultural traditions, as defined there, are much more akin to biological traits than they are to biological (or sociocultural) individuals. They are the sets of behaviours and attributes that pertain to an individual or a group at some level of organization, not the concrete individual or group themselves; in other words, they are features of individuals (such as their behaviour) rather than individuals themselves, and if they are like anything in the biological world, they are traits. To be clear, I do not wish to suggest that traditions are exactly like biological traits – for example, they may well be the result of diffuse mixing, borrowing, and modification across groups. The point is only that they are disanalogous to individual organisms, which makes the proposed analogy problematic. And the ETI approach, standardly construed, is very much about concrete individuals and groups.

What about the social protocell hypothesis and their notion of *socionts* (Andersson & Czárán, 2023)? Similar concerns apply:

The SPH [social protocell hypothesis] follows a familiar evolutionary pathway that is widely considered to have been responsible for most or even all other unequivocal gains in adaptive complexity in natural history … namely an evolutionary transition in individuality … where a new group-level unit of selection (or ‘evolutionary individual’) arises. In this case, the new unit of selection (termed a ‘sociont’) would consist of integrated and adapted cultural lifestyles, coextensive with (but *not* identical to) the social limits of underpinning hominin communities.

In this case, although the evolutionary individuals are taken to be social units, some issues persist. One is that the authors are actually not totally clear on whether socionts are indeed just social units or ‘integrated and adapted cultural lifestyles’ and thereby much more akin to ‘integrated groups of cultural traditions’. Another is that even if we take them to really be social units, there is no obvious reason to think that they should also be reproducers. Moreover, the demographic scale of these socionts is left underspecified. If they are taken to be forager-band scale, they are not sealed units, because individuals and their ideas readily move between groups; if they are ethnolinguistic-scale units, they lack the organizational cohesion needed to act as units of selection. This ambiguity reinforces the general point of the present paper. And thus, the main criticism applies.

All in all, it seems that whether one wishes to find higher-level individuals in social groups or integrated cultural traits of some kind, one has to deviate from the standard and precise formulation of the ETI approach.

## 4. Major evolutionary transitions in sociocultural evolution?

### 4.1. Particular explanations for a particular domain

Does all this mean that we should give up on bringing the MET framework to sociocultural evolution altogether? After all, the MET framework is quite a lot broader than the ETI approach, and sociocultural evolution really does seem to have features that make it amenable to being studied under this framework. These features include (but are likely not exclusive to) the emergence of multilevel organization, division of labour, historical changes of great impact, and some relationship between size, complexity, and autonomy. In other words, we might still think that the commonalities of phenomena between biological and sociocultural evolution outlined earlier on in this paper come down to commonalities of evolutionary processes across the two domains. I surmise that there is room for thinking about sociocultural evolution in MET terms. But the foregoing discussion should demonstrate that we need to be philosophically careful in doing so. We should also bear in mind that despite its predominance the ETI approach is not the only possible approach within this broader framework. And perhaps most importantly, we must make sure that whatever approach we wish to apply to sociocultural evolution should be tailored to that domain. Important similarities should not blind us to important differences, and we saw this clearly when it comes to the role of reproductive selection in biological evolution compared to its virtual absence in sociocultural evolution. Not recognizing this key difference plausibly lies at the heart of the key issues with CGS (on this also see Chellappoo, 2021).

So, what might be the most pertinent explanatory tools that can be deployed to make sense of the features reminiscent of biological METs mentioned above? One set of candidates includes formal and informal decision theoretic or game theoretic models based on notions like interest alignment or optimality. This is a common underlying theme across work in this area (Davison et al., 2021; Keasar et al., 2023; Townsend et al., 2023), which is unsurprising not just because such models lie at the heart of the ETI approach (Michod, 2000), but also because there is a fairly clear sense in which humans and perhaps even groups of humans are bearers of interests, even if these do not directly translate to fitness interests for groups of humans due to reasons discussed in Section 3. In other words, even if CGS is not reproductive selection and therefore cultural groups generally lack reproductive-fitness interests, they may and plausibly do still have growth-fitness interests, and this may in turn play some explanatory role for them. Investigating precisely what kind of explanatory role this might be and how it might work and interact with other explanantia of group-level adaptations in sociocultural evolution could be a promising area of research.

Relatedly, one might ask what kinds of processes underpin the cohesiveness of sociocultural groups (e.g. particular belief systems, shared language, etc.) or how and why division of labour arises in this domain, in what ways are these similar to and different from their counterparts in biological evolution, and whether there are processes of these sorts that are unique to the sociocultural domain. A candidate of this sort of process is what Graeber and Wengrow call schismogenesis (Graeber & Wengrow, 2021), which is the process whereby members of a cultural group collectively and consciously differentiate between themselves and members of a neighbouring cultural group by deliberately and selectively rejecting the latter’s beliefs or practices (although the more general notion of two close groups differentiating from one another dates back further; Barth, 1969). Another potential candidate is Turchin’s hypothesis discussed above, namely that military innovations have enabled larger political entities (Turchin, 2016). These are in part questions for social scientists but are also relevant to the broader question of whether and how the MET framework might be utilized in the sociocultural domain. A set of potentially even more interdisciplinary questions include those pertaining to the role that ecological factors have played in bringing about episodes of dramatic change – *major transitions* in the more historical sense – in sociocultural evolution. Explicitly posing such questions within the context of the MET framework, while being as clear as possible about the limits of specific approaches within this framework developed for biological evolution (e.g. the ETI approach), should be the way forward if we wish to make better sense of why sociocultural evolution is at the same time similar to yet distinct from biological evolution.

### 4.2. Complexity and autonomy in sociocultural evolution

At the end of Subsection 3.1 I promised to further motivate the main argument of this paper and further defend the claim that a loose formulation of the ETI approach applied to sociocultural evolution is undesirable. I will now argue that this is because it comes with the risk of too easily accepting a particular prediction of this approach.

Yohay Carmel, in his paper discussed briefly above (Carmel, 2023), makes four predictions based on the hypothesis that human societies have undergone an ETI. The first of these is of interest here:

[T]he level of *regulation and control* that society exerts over its members should increase. ETIs always begin with independent organisms that combine into a novel collective entity; the newly emerging entity initially has very little control over its constituents … As the transition progresses, control by the higher level entity over its lower level units may have strong selective advantages … Hence, I predict that, as the sociocultural ETI progresses, societal control over its members will rise correspondingly. The reverse, reduced control, is expected to emerge not gradually, but rather suddenly, propelled by a collapse, such as may occur following take-over by other societies, internal splintering, or severe natural disasters.

One could be forgiven for thinking that this prediction of an inverse relationship between complexity of the higher-level unit and autonomy of lower-level units, in its broad contours, is new. But it is not. In fact, Graeber and Wengrow have recently shown that it can be traced back to the Victorian era (Graeber & Wengrow, 2021), when an emerging intellectual tradition of early anthropologists and sociologists, who argued that as societies evolved towards greater organization and hierarchy, individual freedoms or autonomy within smaller groups tended to decline. Figures like Herbert Spencer and Lewis H. Morgan posited that societal progress entailed increased structuring and specialization, often consolidating power within centralized institutions – thus restricting autonomy at the individual or local level.

Yet Graeber and Wengrow challenge the assumption that social complexity must necessarily reduce individual autonomy. They observe that pre-state societies and early civilizations frequently developed sophisticated social systems without resorting to the same degree of top-down control (most notably the earliest cities in Mesopotamia, the Indus Valley, and Ukraine). This contrast implies that the dynamic Carmel identifies in sociocultural ETIs – where complexity drives increased control – is perhaps more contingent than inevitable. Historical evidence suggests that societies have, at times, balanced complexity with autonomy, shaped by cultural traditions, environmental contexts, and other influences. Graeber and Wengrow’s analysis thus offers an alternative perspective: social complexity does not inherently entail greater control over individuals if other organizational forms are considered viable. And although it is not immediately clear if increasing complexity should necessarily lead to central control even in cases of biological ETIs, the perceived connection between the two, as we have seen, is often invoked.

Carmel, like many others, is drawing on a theoretical approach developed in biology and tentatively applying it to sociocultural evolution to make this prediction. And while I do not wish to make the indefensible claim that the prediction is necessarily going to fail (after all, it is an empirical prediction) it still does mean that we need to be more cautious about how easily we are swayed by empirical support for this prediction – in other words, we need to hold this prediction to particularly high standards of research. This is because not doing so (i.e. accepting it too easily) comes with a degree of social risk: if the loss of individual autonomy is a natural and inevitable consequence of increasing social complexity, then we as individuals should just acquiesce to it (or attempt to live as foragers on a planet that cannot possibly support that mode of life for billions of people). This is precisely the line of argument that Victorian thinkers such as Spencer had taken up and, as Graeber and Wengrow take pains to point out, remains highly pervasive to this day. Importantly, it is dangerous because it can be self-fulfilling and can be used to justify and bring about social injustice. And this is why we should be very careful in how we utilize biological theory in sociocultural evolution, particularly the MET framework and the ETI approach.

## 5. Conclusion

This paper has critically examined the applicability of the Evolutionary Transitions in Individuality framework to sociocultural evolution. Although human societies undoubtedly exhibit features such as multilevel organization, cooperation, and division of labour – features often associated with ETIs – these similarities do not extend to the core explanatory conditions of the ETI approach. In particular, the absence of autonomous reproduction at the level of social groups, the lack of reproductive division of labour, and the dominance of growth- and stability-based modes of selection over reproductive selection together suggest that human societies do not form Darwinian populations in the relevant sense. This undermines the claim that they are higher-level evolutionary individuals in the biological sense.

Attempts to apply the ETI framework to the sociocultural domain often rely on weakening or reinterpreting its central criteria. But as I have argued, doing so compromises the very features that make ETI theory theoretically coherent and explanatorily powerful. If the conditions for individuality are made too flexible, the framework risks losing its capacity to distinguish between genuine evolutionary transitions and more ordinary cases of social or institutional complexity. A further risk is that applying the ETI framework too readily to sociocultural evolution diverts attention from alternative explanatory strategies, including ones not rooted in biological theory. Human history may in fact exemplify a pathway to large-scale social complexity that does not depend on ETIs at all, and recognizing this possibility could broaden the search for other, perhaps currently overlooked, routes to complex organisation. Moreover, as McShea has stressed, we lack systematic data that would be needed to test such claims in the first place, which makes the speculative extension of the ETI framework even less compelling. Although the ETI approach remains indispensable for understanding certain episodes in biological evolution, its direct extension to human sociocultural history is not just empirically questionable – it is conceptually misguided. Recognizing this should not lead us to abandon evolutionary explanation in this domain, but rather to seek out approaches better suited to its distinctive dynamics.

## Data Availability

N/A.

## Research transparency and reproducibility interest

N/A.
